# Subtractive modification of bacterial consortium using antisense peptide nucleic acids

**DOI:** 10.3389/fmicb.2023.1321428

**Published:** 2024-01-08

**Authors:** Tatsuya Hizume, Yu Sato, Hiroaki Iwaki, Kohsuke Honda, Kenji Okano

**Affiliations:** ^1^Department of Biotechnology, Graduate School of Engineering, Osaka University, Osaka, Japan; ^2^Division of Life Science, Graduate School of Sciences and Technology for Innovation, Yamaguchi University, Yamaguchi, Japan; ^3^Department of Life Science and Biotechnology, Faculty of Chemistry, Materials and Bioengineering, Kansai University, Osaka, Japan; ^4^International Center for Biotechnology, Osaka University, Osaka, Japan; ^5^Industrial Biotechnology Initiative Division, Institute for Open and Transdisciplinary Research Initiatives, Osaka University, Osaka, Japan

**Keywords:** peptide nucleic acid, cell penetrating peptide, microbiome engineering, antisense effect, growth linkage analysis

## Abstract

Microbiome engineering is an emerging research field that aims to design an artificial microbiome and modulate its function. In particular, subtractive modification of the microbiome allows us to create an artificial microbiome without the microorganism of interest and to evaluate its functions and interactions with other constituent bacteria. However, few techniques that can specifically remove only a single species from a large number of microorganisms and can be applied universally to a variety of microorganisms have been developed. Antisense peptide nucleic acid (PNA) is a potent designable antimicrobial agent that can be delivered into microbial cells by conjugating with a cell-penetrating peptide (CPP). Here, we tested the efficacy of the conjugate of CPP and PNA (CPP-PNA) as microbiome modifiers. The addition of CPP-PNA specifically inhibited the growth of *Escherichia coli* and *Pseudomonas putida* in an artificial bacterial consortium comprising *E. coli*, *P. putida*, *Pseudomonas fluorescens*, and *Lactiplantibacillus plantarum*. Moreover, the growth inhibition of *P. putida* promoted the growth of *P. fluorescens* and inhibited the growth of *L. plantarum*. These results indicate that CPP-PNA can be used not only for precise microbiome engineering but also for analyzing the growth relationships among constituent microorganisms in the microbiome.

## 1 Introduction

A variety of microorganisms have been isolated from the natural environment over the past two centuries. The elucidation of their biological functions and industrial applications has long been a subject of research interest by microbiologists. However, in nature, microorganisms do not exist alone; rather, they are part of a huge ecosystem formed by interactions among microorganisms. These complex microbial systems are known as microbiomes and have a profound impact on the physiology of their host and the state of their habitat. For example, in the human gut, gut microbiome benefits host health by providing protection against pathogens (Chiu et al., 2017), immunomodulation (Zheng et al., 2020), and nutrient metabolism (Silva et al., 2020). Conversely, an imbalance in the gut microbial community is involved in the pathogenesis of many diseases such as nonalcoholic fatty liver disease (Wang et al., 2016), colorectal cancer (Sobhani et al., 2019), and type 2 diabetes (Pedersen et al., 2016). Therefore, understanding and controlling the functions of microbiomes is a new frontier in microbiology.

Microbiome engineering provides a possible solution to improve the imbalance in the microbial population and modulate the function of the microbiome. Microbiome engineering aims to manipulate the composition and function of microbes in the microbiome, and many strategies have been proposed to achieve this approach. The oral administration of probiotics can inhibit the growth of disease-associated bacteria and promote host health (Kerry et al., 2018). In addition to live microorganisms, several additives, including feed enzymes (Kiarie et al., 2013), signaling molecules (Vincent et al., 2022), and organic acids (Dai et al., 2021), have been used to promote or inhibit the growth of beneficial or harmful microorganisms. These additives commonly act on a wide range of microorganisms, making it difficult to precisely manipulate the microbiome community structure. In contrast, bacteriophages, bacterial viruses, have attracted attention as precise antimicrobials. The administration of lytic phages to gnotobiotic mice, which were colonized with defined commensal human gut bacteria, reduced the number of susceptible bacteria by one to two orders of magnitude (Hsu et al., 2019). In addition, the use of the engineered temperate phage expressing programmable nuclease-deactivated Cas9, dCas9, enabled gene modulation of its targeted strain, showing the possibility of strain-specific gene modulation in microbiomes (Hsu et al., 2020). These phage-based technologies assume that phages infecting their hosts are available; however, most microorganisms in microbiomes remain uncultured (Steen et al., 2019), limiting their application to microbiome engineering.

Peptide nucleic acids (PNAs) have application potential as alternative, precise antimicrobials. PNA is a bio-mimic of DNA, and its nucleobases are attached to the *N*-(2-aminoethyl)-glycine backbone instead of the sugar-phosphate backbone (Good and Nielsen, 1998). This unnatural structure makes PNA resistant to proteinases and nucleases (Demidov et al., 1994). Considering that PNA can bind RNA and form PNA/RNA heteroduplexes, antisense PNA, which can bind to the mRNA of an essential gene, can inhibit the translation of mRNA and act as an antimicrobial. However, the application of PNA is hindered by its poor uptake by bacterial cells. To overcome this limitation, chemical conjugation of PNA to a variety of carrier compounds has been proposed. Równicki et al. (2017) synthesized a conjugate of vitamin B_12_ and PNA, which was successfully taken up by *Escherichia coli* and *Salmonella* Typhimurium cells via the vitamin B_12_ uptake pathway. Liu et al. (2023) also synthesized a conjugate of glucose polymer and PNA, and the conjugate was delivered into the *E. coli* and *Staphylococcus aureus* cells through the ABC sugar transporter pathway. Among these carriers, cell-penetrating peptides (CPP), which are short cationic peptides and facilitate the cellular uptake of biomolecules, has been used for the longest time and applied to many microorganisms. KFFKFFKFFK ((KFF)_3_K) is a well-known CPP that can permeate the cell membranes of a variety of microorganisms, including Gram-negative and Gram-positive bacteria (Hatamoto et al., 2009; Ghosal and Nielsen, 2012; Bai et al., 2012b; Mondhe et al., 2014). Good et al. (2001) synthesized a CPP and PNA conjugate (CPP-PNA) with a CPP sequence of (KFF)_3_K, and the PNA sequence was designed to bind to the mRNA encoding the acyl carrier protein (*acpP*), an essential protein for bacterial growth. The addition of CPP-PNA targeting *acpP* in *E. coli* successfully inhibited the growth of *E. coli* (Good et al., 2001). This CPP-PNA was further shown to inhibit the growth of the pathogenic *E. coli* strain (Popella et al., 2022). Likewise, a variety of antisense CPP-PNAs against clinically pathogenic bacteria were synthesized, and their antibacterial potency has been validated (Wojciechowska et al., 2020). Importantly, CPP-PNAs can be designed if the genome sequence of the target microorganism is available, and can be synthesized by solid-phase synthesis (Pipkorn et al., 2012). Therefore, CPP-PNA could be used to modify the microbiome regardless of whether the target microorganism is culturable. However, PNA-based antimicrobials have primarily been applied to purely cultured strains, and few studies have applied them to microbiome engineering.

**Figure 1:**
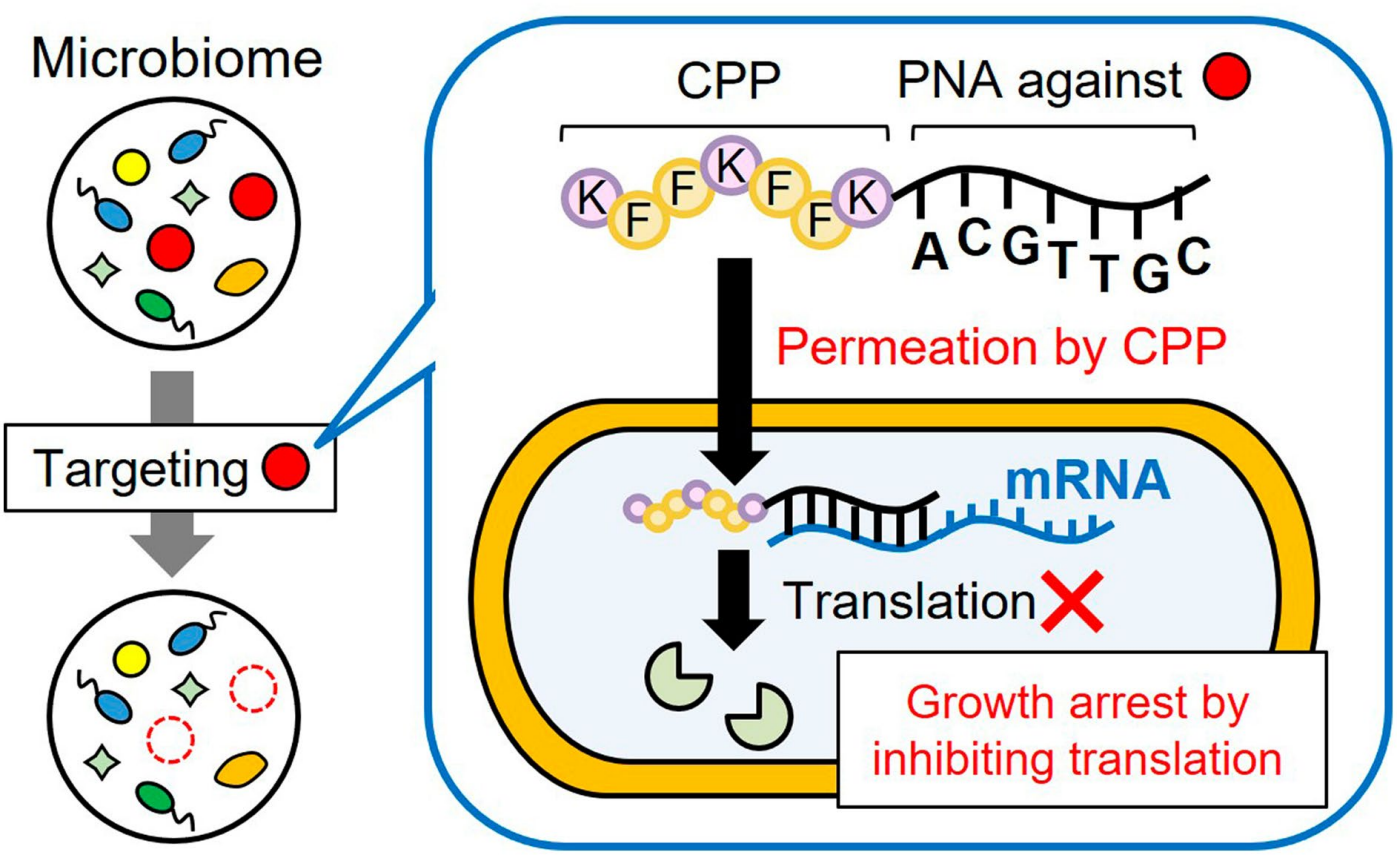
Subtractive modification of the microbiome using a conjugate of CPP and antisense PNA. CPP-PNA can nonspecifically permeate the bacterial membrane in the microbiome through the action of CPP, whereas the PNA region can specifically bind to the mRNA of essential genes of the target species, thereby inducing growth arrest of the target species. As a result, other microorganisms grow and the relative number of target species decrease.

In the present study, we demonstrated microbiome engineering using antisense CPP-PNA in an artificial bacterial consortium (Figure 1) consisting of *E. coli*, *Pseudomonas putida*, *Pseudomonas fluorescens*, and *Lactiplantibacillus plantarum* (formally known as *Lactobacillus plantarum*). The growth of *E. coli* or *P. putida* was selectively inhibited by adding CPP-PNAs to the essential genes in each species, thereby allowing only three bacterial species to grow. Consequently, the bacterial population of the target microorganisms in the consortium can be modified subtractively. Our results also suggested that this microbiome-modification technique could be applied to analyze the growth linkage among the bacteria in the bacterial community. This analysis was accomplished by evaluating how the growth inhibition of one microorganism alters the growth of others.

## 2 Materials and methods

### 2.1 Bacterial strains

The strains used in this study are listed in Table 1. *E. coli* MG 1655 was obtained from National Institute of Genetics (Shizuoka, Japan). *P. fluorescens* NBRC 15829 and *P. putida* NBRC 14164 were obtained from Biological Research Center, National Institute of Technology and Evaluation (NITE; Tokyo, Japan). *L. plantarum* NCIMB 8826 was obtained from National Collection of Industrial, Food and Marine Bacteria (NCIMB; Scotland, UK). The chloramphenicol-resistant strain of *E. coli* MG 1655 (*E. coli* Cm^R^) and neomycin-resistant strain of *P. putida* NBRC 14164 (*P. putida* Neo^R^) were constructed as described in the Materials and Methods section of the Supplementary material. The resulting *E. coli* Cm^R^ and *P. putida* Neo^R^ in addition to *P. fluorescens* and *L. plantarum* were used for growth-inhibition and microbiome-modification experiments using CPP-PNAs.

**Table 1:** Bacterial strains and CPP-PNAs used in this study.

Strain or CPP-PNA	Relevant description or sequence^a^	Source or reference
Strain		
*Escherichia coli*		
TG1	*supE thi-1* Δ(*lac-proAB*) Δ(*mcrB-hsdSM*)*5* (r_K_^−^ m_K_^−^) [F *traD36 proAB lacIqZΔM15*]	Zymo Research
MG 1655	*F^−^ lambda^−^ ilvG^−^ rfb-50 rph-1*	National Institute of Genetics
		
Cm^R^	MG1655 derivative whose *frmA* gene was replaced with *cat* gene, chloramphenicol resistance	This study
S17-1	*F^−^*, *thi*, *pro*, *hsdR*, [RP4-2 Tc::*Mu* Km::*Tn7* (Tp Sm)]	National Institute of Genetics
		
*Pseudomonas fluorescens*		
NBRC 15829	natural resistance to streptomycin	NITE
*Pseudomonas putida*		
NBRC 14164		NITE
Neo^R^	NBRC 14164 derivative whose *kdsD* was replaced with *kan*, neomycin-resistance	
*Lactiplantibacillus plantarum*		NCIMB
NCIMB 8826	Growth in MRS medium adjusted pH at 6.0	
CPP-PNA		
Free CPP	(H)-KFFKFFKFFK-(NH_2_)	Panagene
CPP-*Ec*PNA	(H)-KFFKFFKFFK-eg1-ctcatactct-(NH_2_)	Panagene
CPP-*Pp*PNA	(H)-KFFKFFKFFK-eg1-cgagctcgaa-(NH_2_)	Panagene

^a^eg1, ethylene glycol linker.

### 2.2 CPP-PNA

The sequences of CPP-PNAs used in this study are listed in Table 1. CPP-PNAs were synthesized by Panagene Inc. (Daejeon, South Korea) with a purity of 99.9% and their target sequences are shown in Table 2. CPP-*Ec*PNA was designed to bind the −5 to +5 region of the mRNA of the *acpP* gene in *E. coli*, whereas CPP-*Pp*PNA was designed to bind the +4 to +13 region of the mRNA of the *ftsZ* gene in *P. putida*. CPP with a (KFF)_3_K sequence was attached to the N-terminal of both PNAs via an ethylene glycol linker. The CPP-PNAs were obtained as dry pellets and dissolved in presterilized deionized water to give 50 μM. The samples were incubated at 90°C for 10 min immediately before use. To ensure the sequence specificity of CPP-*Ec*PNA and CPP-*Pp*PNA for the target region of the mRNA, off-target analysis was performed by comparing the target sequences of CPP-PNAs with the genome sequences of *E. coli* (NCBI RefSeq: NC_000913.3), *P. fluorescens* (NCBI RefSeq: NZ_BDAA00000000.1), *P. putida* (GenBank: AP013070.1), and *L. plantarum* (GenBank: AL935263.2).

**Table 2:** Off-target analysis of PNA in four bacterial strains.

Target sequence of PNA			Number of off-targets in the genome^a^				Off-targets overlapping translation initiation region^a,b^			
Species	Gene	Sequence	*Ec*	*Pp*	*Pf*	*Lp*	*Ec*	*Pp*	*Pf*	*Lp*
*E. coli*	*acpP*	AGAGTATGAG	11	4	3	4	0	0	0	0
*P. putida*	*ftsZ*	TTCGAGCTCG	1	19	13	4	0	0	0	0

^a^*Ec*, *Pp*, *Pf*, and *Lp* indicate *E. coli*, *P. putida*, *P. fluorescens*, and *L. plantarum*, respectively.

^b^ − 16 to + 6 region from translation start site.

### 2.3 Evaluation of growth inhibition by using CPP-PNAs

*Escherichia coli* Cm^R^, *P. fluorescens*, and *P. putida* Neo^R^ were cultivated in Luria–Bertani (LB) medium at 30°C, whereas *L. plantarum* was cultured in 5 mL of MRS medium (Difco Laboratories, Detroit, MI, USA) at 30°C. Then, 50 μL aliquots of each culture were mixed with an equivalent volume of 30% (w/v) glycerol solution and stored at −80°C until use.

For the precultivation of *E. coli*, *P. fluorescens*, and *P. putida*, M9 minimal medium (glucose, 4 g/L; Na_2_HPO_4_, 6 g/L; K_2_HPO_4_, 3 g/L; NaCl, 0.5 g/L; NH_4_Cl, 1 g/L; MgSO_4_, 1 mM; CaCl_2_, 0.3 mM; thiamine∙HCl, 1 mg/L) was used, whereas MRS medium diluted 10 times was used for precultivation of *L. plantarum*. One portion of the glycerol stock was inoculated into a test tube containing 5 mL of the medium and cultivated at 30°C for 14 h. The cells were collected by centrifugation at 8,000 × *g* at 4°C for 3 min and washed twice with 2 mM PIPES-NaOH (pH = 6.8). Then, the cells were resuspended in the same buffer to a cell concentration of 1.0 × 10^7^ CFU/mL.

The main cultivation was performed in a 1.5-mL PROKEEP low-binding microtube (Fukae-Kasei Co., Ltd., Hyogo, Japan) to evaluate the growth inhibitory effect of the CPP-PNAs on each of the four bacteria. Each of the four strains was inoculated into microtubes containing 200 μL of the medium as in the pre-culture at 1.0 × 10^5^ CFU/mL, and each CPP-PNA was added at 0–10 μM. The microtubes were sealed with Parafilm M (Sigma-Aldrich Co. LLC, St. Louis, MO, USA) and cultivated at 30°C and 180 rpm. After 24 h of cultivation, the culture was serially diluted and 50 μL of dilutants were spotted on the agar media. In determining viable cell numbers of *E. coli*, *P. fluorescens*, and *P. putida*, LB media supplemented with 30 μg/mL of chloramphenicol, 50 μg/mL of streptomycin, and 50 μg/mL of neomycin were used, respectively. The MRS medium prepared at pH 6.0 was used for the cultivation of *L. plantarum*. *E. coli* and *L. plantarum* were cultivated at 37°C, whereas *P. fluorescens* and *P. putida* were cultivated at 30°C.

### 2.4 Subtractive modification of bacterial consortium using CPP-PNAs

A cell suspension (1.0 × 10^7^ CFU/mL) of *E. coli* Cm^R^, *P. fluorescens*, *P. putida* Neo^R^, and *L. plantarum* was prepared as described above. The aforementioned strains were co-inoculated at 1.0 × 10^5^ CFU/mL onto each microtube containing 200 μL of M9 medium to form artificial bacterial consortium. Then, CPP-*Ec*PNA or CPP-*Pp*PNA were added to the microtubes to inhibit the growth of their target bacteria, *E. coli* or *P. putida*, respectively. The bacterial consortium was cultivated at 180 rpm and 30°C, and the cultures were regularly harvested. Finally, the consortium was serially diluted, and 50 μL of dilutant was spotted on four different agar media as described above to selectively cultivate and count the colony number of each of the four strains.

## 3 Results

### 3.1 Selection of microbes to constitute the artificial bacterial consortium

The natural microbiome is composed of a mixture of Gram-negative and Gram-positive bacteria from a variety of genera and species. In this study, *E. coli*, *P. putida*, *P. fluorescens*, and *L. plantarum* were selected to construct the artificial bacterial consortium, and *E. coli* and *P. putida* were used as the target bacteria to induce species-selective growth inhibition. This model is suitable for elucidating whether PNA can be used for subtractive modification of microbiome, including bacteria across (1) species, (2) genera, and (3) Gram-negative and Gram-positive bacteria. In co-culture system, it is difficult to individually monitor the growth of the constituent microorganisms. From this point of view, this bacterial combination is useful as it allows to individually evaluate viable cell numbers using the corresponding selective media for each microorganism (see the Materials and methods section). *P. fluorescens* is naturally resistant to streptomycin, and *L. plantarum* grew in the MRS medium, with pH adjusted to 6.0 (Supplementary Table 1), whereas other bacteria hardly grew under these conditions. *E. coli* and *P. putida* are the representative hosts for genetic modification. Thus, the antibiotic-resistant derivatives, *E. coli* Cm^R^ and *P. putida* Neo^R^, were easily constructed (Supplementary materials and methods). Although *P. fluorescens* was resistant to chloramphenicol, it could not grow at 37°C. Therefore, *E. coli* could be selectively grown on LB medium supplemented with chloramphenicol at 37°C.

### 3.2 Selection of CPP-PNAs for species-specific growth inhibition

The antibacterial activity of PNA is largely affected by various factors, such as the target gene, localization of the target sequence, and PNA length (Goltermann et al., 2019). Therefore, we selected PNAs, which are already known to have antibacterial activity, to facilitate the proof-of-concept of microbiome engineering using CPP-PNAs. mRNAs of several essential genes were targeted by CPP-PNAs in *E. coli* such as *acpP* (Good et al., 2001), *rpoD* (Bai et al., 2012a), and *murA* (Mondhe et al., 2014). Among them, CPP-PNA, which targets mRNA of *acpP* (CPP-*Ec*PNA) showed the lowest minimal inhibitory concentration (MIC) of 0.6 μM in *E. coli* (Wojciechowska et al., 2020). For the growth inhibition of pseudomonads, only *Pseudomonas aeruginosa* has been targeted by antimicrobial PNA because this strain causes infectious diseases. In *P. aeruginosa* PAO1, mRNAs of *acpP* and *ftsZ* genes were targeted by antisense CPP-PNAs, and both CPP-PNAs had the same MIC of 2.0 μM (Ghosal and Nielsen, 2012). Remarkably, the target sequence of *ftsZ* showed mismatches among species, with four different sequence variations among the six species (Supplementary Table 2). In contrast, the target sequence of *acpP* showed lower diversity, and four species had the same sequence as that of *P. aeruginosa*. Therefore, CPP-PNA, which targets *ftsZ* (CPP-*Pp*PNA), was used in this study because of its ability to induce cell death in a species-selective manner, even in the presence of microorganisms belonging to the same genus.

As a CPP, a (KFF)_3_K synthetic peptide was selected and conjugated with PNA because of its ability to transport PNA to Gram-negative bacteria, such as *E. coli*, *P. aeruginosa*, and *Klebsiella pneumoniae*, as well as Gram-positive bacteria, such as *Bacillus subtilis*, *S. aureus*, and *Corynebacterium efficiens* (Hatamoto et al., 2009; Ghosal and Nielsen, 2012; Bai et al., 2012b; Mondhe et al., 2014).

### 3.3 Off-target analysis of CPP-PNAs

In performing microbiome engineering using CPP-PNAs, off-target effects should be minimized. Thus, the potential off-target sequences of CPP-PNAs were analyzed. The comparison of the target sequences of CPP-*Ec*PNA and CPP-*Pp*PNA with the genomes of *E. coli*, *P. putida*, *P. fluorescens*, and *L. plantarum* revealed the presence of several identical sequences. For CPP-*Ec*PNA, 11, 4, 3, and 4 off-targets were found in the genome of each of the four bacteria, whereas 1, 19, 13, and 4 sequences were found for CPP-*Pp*PNA, respectively (Table 2). Dryselius et al. (2003) reported that the translation initiation region, including the ribosome-binding site and start codon, is sensitive to antisense PNA inhibition. Analysis of 2,458 bacterial genomes revealed that the commonly utilized ribosome-binding sequences have a length of 3–6 nt and were 5–10 nt away from the start codon (Omotajo et al., 2015). Moreover, the +4 to +6 region from the translation start site showed sensitivity to antisense PNA inhibition (Ghosal and Nielsen, 2012). Therefore, the PNA overlapping the −16 to +6 region will show a high antimicrobial effect. None of the off-target sequences was located in this region of any gene (Table 2). Thus, CPP-*Ec*PNA and CPP-*Pp*PNA are expected to show high specificity for their respective target sequences.

### 3.4 Evaluation of the antibacterial activity of CPP-PNAs

**Figure 2:**
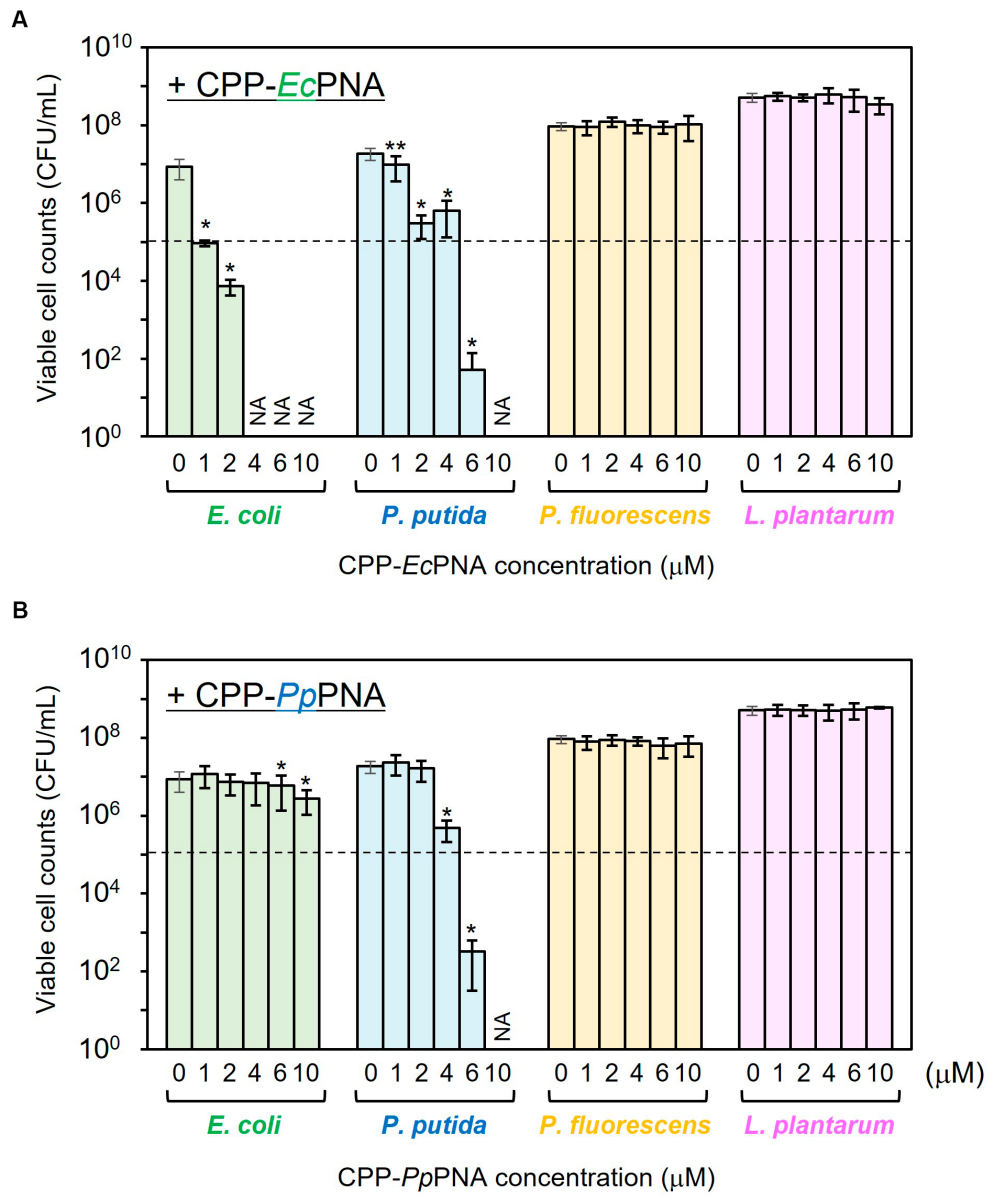
Evaluation of the antibacterial activity of CPP-PNAs against four bacterial species. CPP-*Ec*PNA **(A)** or CPP-*Pp*PNA **(B)** was added at various concentrations to the culture of *E. coli*, *P. putida*, *P. fluorescens*, and *L. plantarum*. After cultivation at 30°C for 24 h, the viable cell numbers were counted. Dashed line indicates the initial cell concentration prior to cultivation and NA indicates that growth inhibitory effect was not assessed. Data bars represent mean ± standard deviation values of three independent experiments. The growth of each strain with the addition of CPP-PNAs was compared with that in nontreated condition. Asterisks and double-asterisks indicate *p* values are less than 0.05 and 0.01 in the *t*-test, respectively.

The antibacterial activities of CPP-*Ec*PNA and CPP-*Pp*PNA against the four bacterial species were evaluated. When *E. coli* was cultivated in the absence of CPP-PNAs, the number of viable cells increased from 1.00 × 10^5^ CFU/mL to 8.60 × 10^6^ CFU/mL after 24 h of cultivation (Figure 2A). On the other hand, no growth of *E. coli* was observed in the presence of 1 μM CPP-*Ec*PNA and the number of viable cells after 24 h of cultivation was 9.27 × 10^4^ CFU/mL (*p* = 0.044 in *t*-test). For the other bacterial species, *Ec*PNA can be considered as a scrambled PNA with random PNA sequences. No significant decrease in the viable cell number was observed in the nontarget species, *P. fluorescens* and *L. plantarum*, even in the presence of 10 μM CPP-*Ec*PNA (*p* = 0.36 and 0.079, respectively). However, CPP-*Ec*PNA showed toxicity to *P. putida*. The addition of ≥2 μM CPP-*Ec*PNA had a decisive effect on the growth of *P. putida*. In the presence of 2 μM CPP-*Ec*PNA, the number of viable cells dropped from 1.87 × 10^7^ CFU/mL to 2.97 × 10^5^ CFU/mL (98.4% decrease; *p* = 0.018), whereas the addition of 1 μM CPP-*Ec*PNA maintained the growth of *P. putida* at 52.4% (*p* = 0.0013).

CPP-*Pp*PNA showed a slightly higher specificity than CPP-*Ec*PNA (Figure 2B). Although the addition of 4 μM CPP-*Pp*PNA did not completely inhibited the growth of *P. putida* (4.79 × 10^5^ CFU/mL; *p* = 0.018), the addition of 6 μM CPP-*Pp*PNA was enough to inhibit the growth of *P. putida*. The number of viable cells decreased from 1.00 × 10^5^ CFU/mL to 3.20 × 10^2^ CFU/mL after 24 h of cultivation, which is significantly lower than the growth of the strain without adding CPP-*Pp*PNA (1.87 × 10^7^ CFU/mL; *p* = 0.019). At this concentration, no growth inhibition was observed in the nontarget species, *P. fluorescens* and *L. plantarum*. Because the +4 to +13 region of the *ftsZ* gene, which is the target sequence of *Pp*PNA, differs by only one base pair between *P. putida* and *P. fluorescens* (Supplementary Table 2), *Pp*PNA works as an one-mismatched PNA against *P. fluorescens*. This result indicates that one-mismatched PNA is not effective to target *ftsZ* of *P. fluorescens* at this concentration. On the other hand, *E. coli* showed a decrease in its growth, but 69.4% of growth was retained (*p* = 0.021).

It is well known that free PNA poorly penetrate bacterial cell membrane. The addition of free PNA (with the same sequence as in this experiment) at a high concentration of 32 μM did not inhibit the growth of *E. coli* MG1655 (Goltermann et al., 2022). In addition, the addition of 32 μM free PNA targeting RNA polymerase α-subunit (*rpoA*) of *Listeria monocytogens* also showed no growth inhibitory effect (Abushahba et al., 2016), though its CPP conjugates inhibited the growth of *L. monocytogens* at lower concentrations (1–2 μM). On the other hand, some CPPs are known to show cytotoxicity (Lee et al., 2021). To evaluate whether the growth inhibition of *E. coli* and *P. putida* by CPP-PNAs was not due to CPP-induced cytotoxicity, the effect of free CPP on the growth of *E. coli* and *P. putida* was investigated (Supplementary Figure 1). In the presence of 6 μM free CPP, the number of viable cells of *E. coli* and *P. putida* increased to 8.73 × 10^6^ CFU/mL and 2.90 × 10^7^ CFU/mL after 24 h of cultivation, respectively. These values are comparable to those of nontreated cells (1.65 × 10^7^ CFU/mL and 2.15 × 10^7^ CFU/mL, respectively) and no significant decrease was observed (*p* = 0.13 and 0.29, respectively). Therefore, we concluded that the growth inhibition of *E. coli* and *P. putida* was caused by the CPP-PNA conjugates, and 1 μM CPP-*Ec*PNA and 6 μM CPP-*Pp*PNA were used for the following microbiome modifications.

### 3.5 Subtractive modification of artificial bacterial consortium

**Figure 3:**
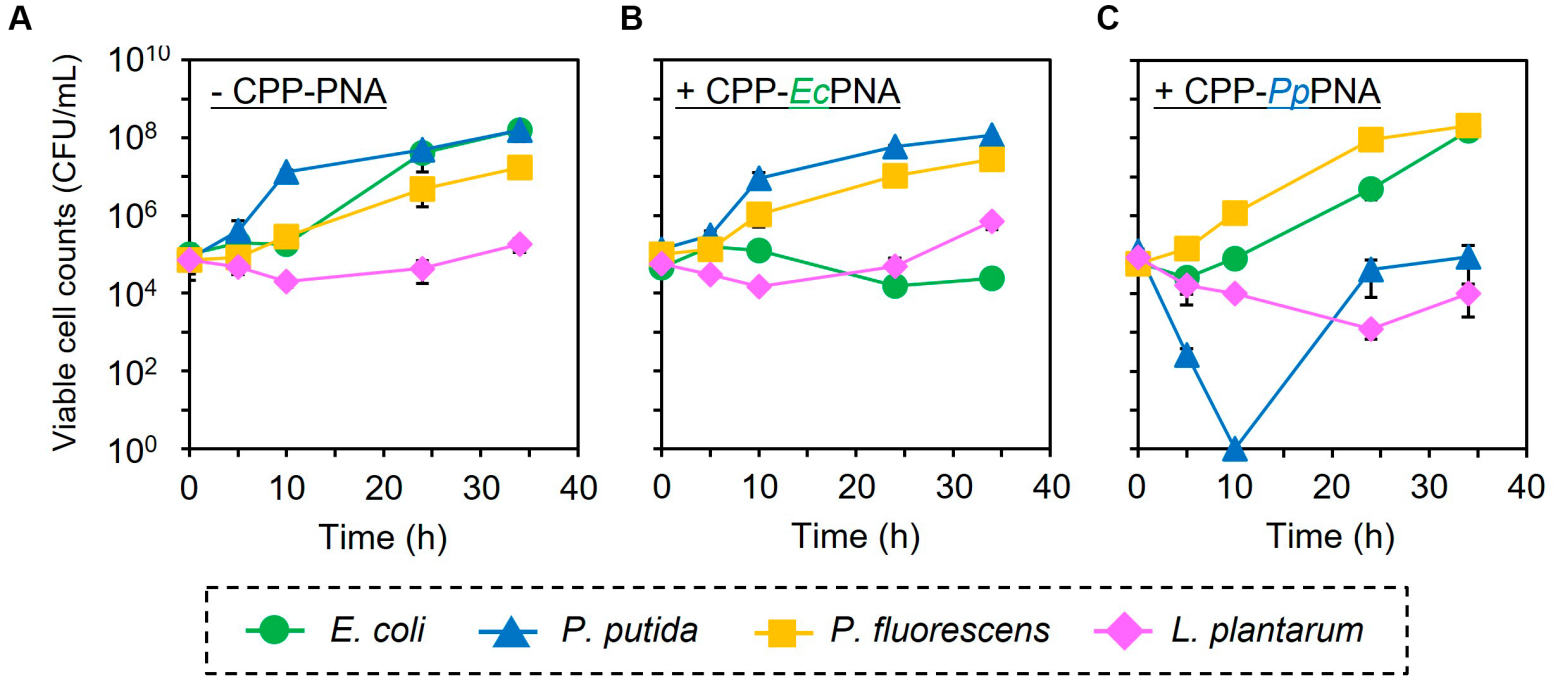
Subtractive modification of the artificial bacterial consortium consisting of four bacterial species. *E. coli* (green circles), *P. putida* (blue triangles), *P. fluorescens* (orange squares), and *L. plantarum* (pink diamonds) were co-inoculated to M9 medium at 1.0 × 10^5^ CFU/mL of each. The growth of four bacteria without CPP-PNAs **(A)**, with CPP-*Ec*PNA **(B)**, and with CPP-*Pp*PNA **(C)** was compared. Data points represent mean ± standard deviation of three independent experiments.

**Figure 4:**
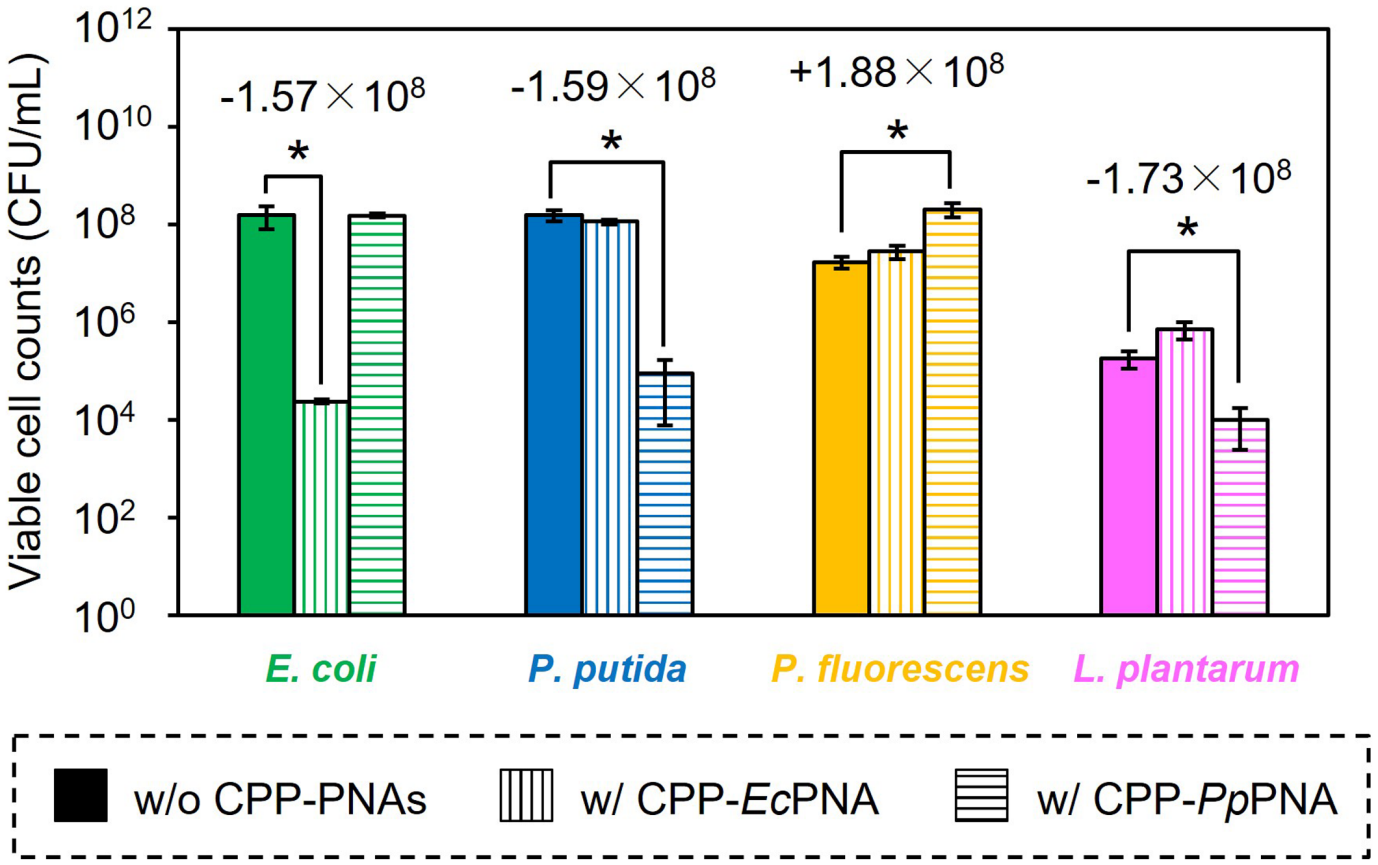
Comparison of the final CFU after modification of the artificial bacterial consortium. *E. coli* (green bars), *P. putida* (blue bars), *P. fluorescens* (orange bars), and *L. plantarum* (pink bars) were co-inoculated to M9 medium and were co-cultivated at 30°C for 34 h without CPP-PNAs (solid bars), with CPP-*Ec*PNA (vertical bars), and with CPP-*Pp*PNA (horizontal bars). Data bars represent the mean ± standard deviation values in three independent experiments. Asterisks indicate *p* values are less than 0.05 in the *t*-test. For the results showing statistical significance, the difference of viable cell counts to the nontreated condition was shown in the figure.

The artificial bacterial consortium was constructed by co-cultivating four bacterial species in M9 minimal medium at an initial cell concentrations of 10^5^ CFU/mL of each. When the bacterial consortium was cultivated without adding CPP-PNAs, numbers of viable cell of *E. coli*, *P. putida*, *P. fluorescens*, and *L. plantarum* reached 1.57 × 10^8^, 1.59 × 10^8^, 1.71 × 10^7^, and 1.83 × 10^5^ CFU/mL after 34 h of cultivation, respectively (Figure 3A). The addition of 1 μM of CPP-*Ec*PNA completely inhibited the growth of *E. coli*, and the viable cell number at 34 h was 2.38 × 10^4^ CFU/mL (Figure 3B; *p* = 0.035). On the other hand, no significant change was observed in the growth of *P. fluorescens* and *L. plantarum* as the *p* values were 0.12 and 0.059 in *t*-test, respectively (Figure 4). Although slight growth inhibition was observed in the monoculture of *P. putida* (Figure 2A), no significant growth inhibition was observed with the addition of CPP-*Ec*PNA in the coculture experiment (*p* = 0.069; Figure 4). Since CPP-*Ec*PNA is taken up by the four bacterial species, the effective concentration of CPP-*Ec*PNA for each of the four bacteria may be lower than that in monoculture experiments. As well as monoculture experiment, addition of higher concentration of CPP-*Ec*PNA (2 μM) inhibited the growth of *P. putida* in microbiome modification (Supplementary Figure 2). About 95% decrease was observed in numbers of viable cell after 34 h of cultivation (8.13 × 10^6^ CFU/mL; *p* = 0.011) compared to those without CPP-*Ec*PNA (1.59 × 10^8^ CFU/mL). Consequently, the growth of *E. coli* was successfully inhibited by adding 1 μM of CPP-*Ec*PNA without perturbing the growth of nontarget bacteria, and the artificial bacterial consortium was subtractively modified.

**Figure 5:**
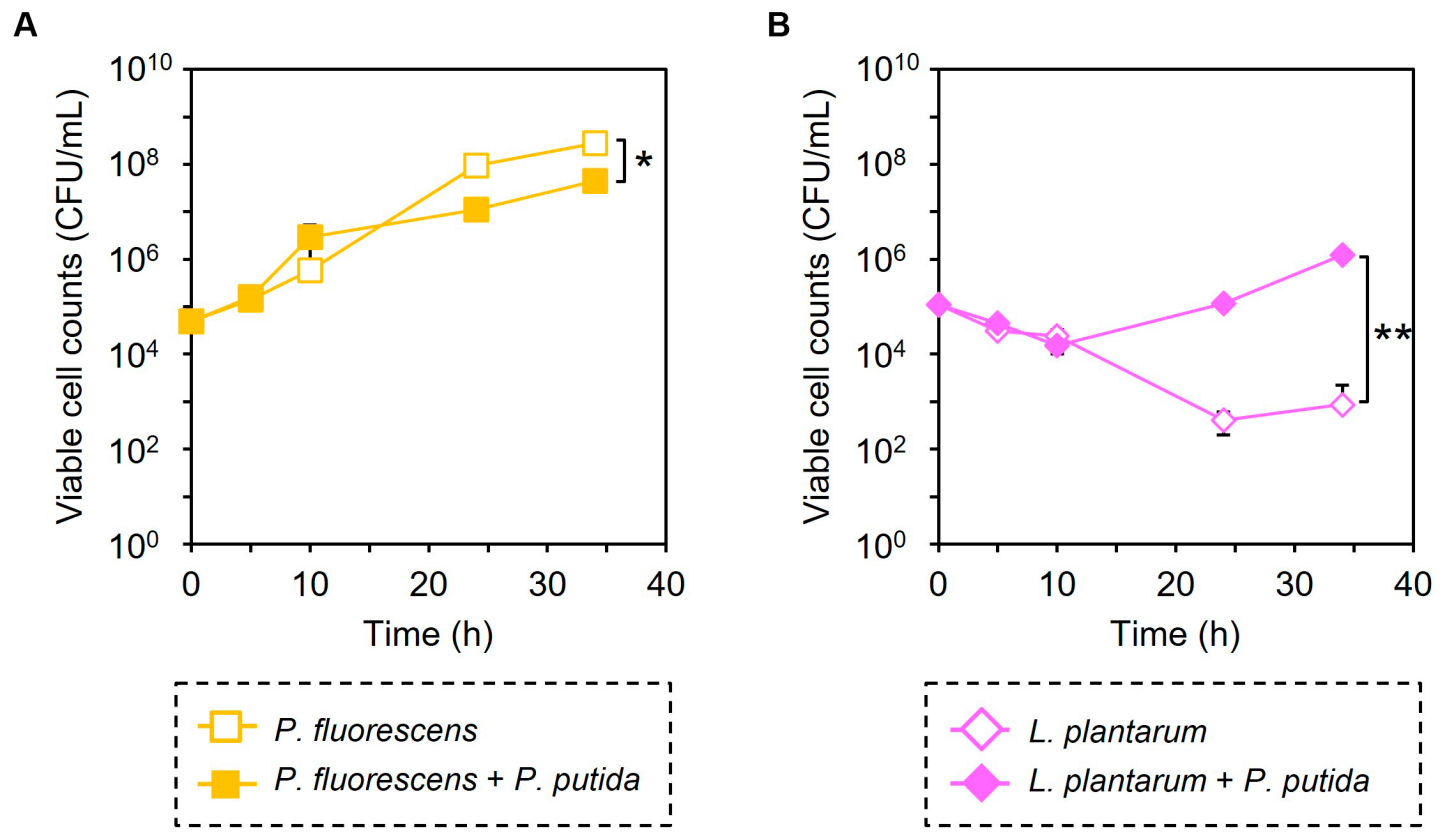
Comparison of bacterial growth with and without *P. putida*. *P. fluorescens* **(A)** and *L. plantarum* **(B)** were cultivated without (open symbols) and with (closed symbols) *P. putida* in M9 medium. Data points represent the mean ± standard deviation values in three independent experiments. Asterisk and double asterisk indicate that the *p* values are less than 0.05 and 0.01 in the *t*-test, respectively.

Similarly, the growth inhibition of *P. putida* was induced by adding 6 μM of CPP-*Pp*PNA, and the viable cell numbers of *P. putida* once decreased to undetectable level (< 20 CFU/mL) after 10 h of cultivation. Then, it increased to 8.93 × 10^4^ CFU/mL after 34 h of cultivation (Figure 3C). To confirm whether this growth recovery was due to the development of PNA-resistant mutant, four colonies were randomly selected from the colonies formed after 34 h of cultivation, and their resistance to CPP-*Pp*PNA was evaluated. As well as the parental strain, the viable cell counts of all four strains were under the detection limit (20 CFU/mL) after 10 h of cultivation in the presence of 6 μM of CPP-*Pp*PNA (Supplementary Figure 3). Therefore, growth restoration of *P. putida* does not seem to be due to the acquisition of PNA resistance. Although the mechanism is unknown at this time, it may be that *P. putida* gradually degrades CPP-*Pp*PNA or CPP-*Pp*PNA was incorporated by other bacteria grown normally, allowing it to grow in the later stages of cultivation. Interestingly, an increase and decrease in the viable cell numbers of *P. fluorescens* and *L. plantarum* were also observed (6.65 × 10^7^ and 9.87 × 10^3^ CFU/mL, respectively), with statistical significance (*p* = 0.023 and 0.025, respectively, Figure 4). In determining whether the changes in the growth of *P. fluorescens* and *L. plantarum* were due to the addition of CPP-*Pp*PNA or not, *E. coli*, *P. fluorescens*, and *L. plantarum* were co-cultivated without adding CPP-*Pp*PNA (Supplementary Figure 4A). These three bacteria showed almost the same growth profiles as those of the four bacterial co-culture systems with the addition of CPP-*Pp*PNA (*p* = 0.42, 0.30 and 0.20, respectively; Supplementary Figure 4B). This result implies that the changes in the growth of nontarget bacteria were not induced by CPP-*Pp*PNA, indicating that *P. putida* inhibited the growth of *P. fluorescens* and stimulated the growth of *L. plantarum* in the four bacterial co-culture systems. This hypothesis was verified by comparing the growth profiles of *P. fluorescens* and *L. plantarum* between the monoculture and co-culture with *P. putida*. The result revealed that *P. putida* inhibited the growth of *P. fluorescens* and stimulated the growth of *L. plantarum* (Figure 5). These results suggested that CPP-PNA is beneficial not only for microbiome engineering but also for revealing growth linkages among microorganisms that make up the microbiome.

## 4 Discussion

To date, CPP-PNAs have been used as an alternative to antibiotics and have focused on inhibiting the growth of pathogenic bacteria (Kulyté et al., 2005; Ghosal and Nielsen, 2012; Bai et al., 2012a) including their antibiotic-resistant mutants such as methicillin-resistant *S. aureus* (Bai et al., 2012b). Most of these studies have examined the target genes and targeting position in the mRNA using PNA, which exhibits high antisense effects, and few reports have examined their effects on the growth of microorganisms other than the target microorganism (Mondhe et al., 2014). In contrast, this study aimed to apply CPP-PNA as a tool for precise microbiome engineering in a subtractive manner. Thus, the selectivity of CPP-PNAs for the target bacteria should be considered. Our results demonstrated that CPP-*Ec*PNA and CPP-*Pp*PNA selectively inhibited the growth of their target bacteria, *E. coli* and *P. putida*, in a four-species mixed culture (Figure 3). To the best of our knowledge such species-specific growth inhibition using antisense PNA has only been reported by Mondhe et al. (2014). In their study, the growth of *K. pneumoniae* and *S.* Typhimurium was selectively inhibited by the addition of CPP-PNA targeting each of them in a three-species mixed culture, including *Bacillus subtilis*. Consequently, CPP-PNA was shown to be used for subtractive modification of the microbiome, including the bacteria across genera, and Gram-negative and Gram-positive bacteria. Our result that CPP-*Pp*PNA selectively inhibited the growth of *P. putida* in the presence of *P. fluorescens* (Figure 3) indicates that CPP-PNA can exert selectivity, even at the species level, by selecting the appropriate PNA sequence.

In addition, PNA-based microbiome engineering could be used to analyze the growth relationships among microorganisms in the microbiome. Microbiome engineering creates the microbiome with the desired functions by artificially modifying the composition and function of microorganisms in the microbiome. However, this is only one aspect of microbiome engineering. Removing a particular microorganism from the microbiome is nothing more than creating a microbiome in which that microorganism is absent. Comparing the behavior and function of the microbiome before and after modification would determine the role of the removed microorganisms and their interactions with other microorganisms in the original microbiome. Our results showed that addition of CPP-PNA to the microbial consortium did not completely eliminate the target species, but reduced the number of the cells from the initial level of 1.00 × 10^5^ CFU/mL (Figure 3). On the other hand, the number of cells of other species increases, allowing the population of the target species to be lowered over time. As a result, the contribution of the target species to the microbial consortium can be minimized. The growth rate is an easy change to detect, and the subtractive modification of microbiome in four bacterial systems successfully reveals that *E. coli* did not interfere with the growth of other microorganisms, but *P. putida* inhibited the growth of *P. fluorescens* and enhanced the growth of *L. plantarum* (Figure 4). As observed in our study, *L. plantarum* can hardly grow in M9 medium, which contains no amino acids and vitamins except for thiamine, without co-cultivating other bacteria (Mizuno et al., 2017), because *L. plantarum* needs a variety of nutrients such as vitamins and amino acids (Wegkamp et al., 2010). Accordingly, *P. putida* must support the growth of *L. plantarum* by providing nutrients. Understanding such a commensalism, as well as other interactions such as mutualism, cooperation, and competition, is necessary to understand the dynamics of the microbiome.

Compared to our top-down approach, several studies have employed a bottom-up approach to analyze the interactions of the constituent microorganisms in the microbiome. Venturelli et al. (2018) selected 12 prevalent human-associated intestinal species and analyzed the growth linkages for 66 combinations of two species. From these datasets, a predictive computational model for the dynamics of the microbial community was developed. Considering that interactions between two species are often modulated by a third species, Bairey et al. (2016) constructed a model that accounted for high-order interactions. The difficulty in applying such techniques to predict the dynamics of “real” microbiome may depend on the large species that comprise the microbiome. It is estimated that at least 160 bacterial species present in the microbiome of each individual (Qin et al., 2010), and experimental assessment of the growth linkages of each microorganism will require a great deal of time and effort. Moreover, the bottom-up approach assumes the use of isolated microorganisms and is not applicable to approximately 70% of the species that have not yet been cultured (Almeida et al., 2021). In contrast, PNA-based subtractive microbiome modification (top-down approach) has the potential to target all bacteria in the microbiota if genome sequences are available. Furthermore, this approach will allow us to examine the impact of one microbial species on the growth of all other species at once by inhibiting the growth of one microbial species.

A key obstacle in using CPP-PNAs to modify the actual microbiome is increasing microbial specificity. In monoculture experiments using four different microorganisms, CPP-*Ec*PNA and CPP-*Pp*PNA showed nonspecific growth inhibition against *P. putida* and *E. coli*, respectively, and inhibition became more pronounced as the concentration of CPP-PNAs increased (Figure 2). Because of this nonspecific growth inhibition, we were unable to add enough CPP-PNAs to completely abolish the target bacteria (Figure 3). In this study, PNAs with 10 nucleobases were used, and there are 1,048,576 variations of the 10 nucleobases (= 4^10^). Given that the bacterial genome size is several Mbp, this PNA variation will not provide sufficient specificity. Therefore, increasing the length of the PNA will increase specificity; however, Goltermann et al. (2019) reported that increasing the length of the PNA decreases the membrane permeation efficiency of the CPP, thereby weakening the antisense effect. One of the advantages of CPPs is their sequence diversity. The CPPs composed of natural amino acids alone can produce 20^n^ (*n* = CPP length) of sequences. Lee et al. (2021) have constructed CPP library including nearly 100 fluorescently labeled CPPs and evaluated their cellular uptake and cytotoxicity. As a result, they succeeded to obtain CPPs suitable for delivery of bioactive cargo into *E. coli*. Finding a CPP with a higher membrane permeation efficiency will allow us to introduce a longer PNA to microorganisms and to modify microbiomes with higher selectivity.

In conclusion, this study demonstrated that CPP-PNAs can selectively inhibit the growth of target microorganisms at the species level, and the model microbiome consisting of the four bacterial species was precisely modified in a subtractive way. This precise microbiome-modification technique will be used to create a model microbiome to prove its function or as a tool for creating synthetic ecosystems with desired functionality. We also demonstrated that PNA-based microbiome engineering could be used to analyze growth relationships among microorganisms in microbiome. Microbial growth linkages provide an opportunity to unravel interactions among microorganisms. By combining various approaches such as metabolomic analysis, it is expected to reveal what interactions are at work between microorganisms and what ecological forces are involved in the assembly and stability of the microbiome.

## Data availability statement

The original contributions presented in the study are included in the article/Supplementary material, further inquiries can be directed to the corresponding author.

## Author contributions

TH: Investigation, Methodology, Writing – review & editing. YS: Investigation, Methodology, Writing – review & editing. HI: Writing – review & editing. KH: Funding acquisition, Supervision, Writing – review & editing. KO: Conceptualization, Formal analysis, Funding acquisition, Investigation, Methodology, Project administration, Writing – original draft.
