# Supplementary material for: Subtractive modification of bacterial consortium using antisense peptide nucleic acids
Source: Front Microbiol. 2024 Jan 8;14:1321428. doi: 10.3389/fmicb.2023.1321428 (PMC10800778; doi:10.3389/fmicb.2023.1321428)
Supplement: Supplementary file 1 [file Data_Sheet_1.PDF]

Supplementary Material

Subtractive modification of bacterial consortium using antisense peptide nucleic acids

Tatsuya Hizume[1], Yu Sato[2], Hiroaki Iwaki[3], Kohsuke Honda[4,5], Kenji Okano[3,4*]

[1]Department of Biotechnology, Graduate School of Engineering, Osaka University, Osaka, Japan

[2]Division of Life Science, Graduate School of Sciences and Technology for Innovation, Yamaguchi University, Yamaguchi, Japan

[3]Department of Life Science and Biotechnology, Faculty of Chemistry, Materials and Bioengineering, Kansai University, Osaka, Japan

[4]International Center for Biotechnology, Osaka University, Osaka, Japan

[5]Industrial Biotechnology Initiative Division, Institute for Open and Transdisciplinary Research Initiatives, Osaka University, Osaka, Japan

*** Correspondence:**
Kenji Okano
okano.k@kansai-u.ac.jp

1 Supplementary Materials and Methods

1.1 Plasmid construction

The plasmids used in this study are summarized in Supplementary Table S3 and the sequences of the oligonucleotide primers used in this study are summarized in Supplementary Table S4. *Escherichia coli* TG1 was purchased from Zymo Research (Irvine, CA, USA) and used as the host strain for plasmid construction. The strain was grown in Luria–Bertani (LB) medium at 37°C. For cultivation of its transformants, 100 μg/mL ampicillin, 100 μg/mL spectinomycin, or 50 μg/mL kanamycin was added to the medium. For the solid media, 1.5% (w/v) agar was added to the medium. PCR was performed using the PrimeSTAR HS DNA polymerase (Takara Bio Inc., Shiga, Japan).

Prior to the construction of the plasmid to replace the *frmA* gene in the genome of *E. coli* MG 1655 with the *cat* gene, a donor DNA cassette for genome editing was constructed using pUC19 as a vector. The 500-bp upstream and downstream sequences of *frmA* were amplified by PCR from the genome of *E. coli* MG 1655 using the primer sets *frmA* up_F/*frmA* up_R and *frmA* down_F/*frmA* down_R, respectively. The amplicons were digested with SalI and ligated together. The ligated fragment was amplified by PCR using primers *frmA* up_F and *frmA* down_R. The amplicon was digested with HindIII and EcoRI, and then inserted into the same restriction enzyme sites of pUC19 (Takara Bio Inc.), resulting in pUC19-Δ*frmA*. Subsequently, the *cat* gene, including the 104-bp upstream (promoter) region, was amplified by PCR from pHLA (Narita et al., 2006) using the primers

*cat*_F and *cat*_R. The amplicon was digested with SacI and SalI, and inserted into pUC19-Δ*frmA*, resulting in pUC19-*frmA*::*cat*. The genome-editing plasmid was constructed using pTargetF, which was a gift from Sheng Yang (Addgene plasmid #62226; http://n2t.net/addgene:62226; RRID:Addgene_62226) (Jiang et al., 2015). The original N_{20} sequence of pTargetF was replaced with that for targeting *frmA* by inverse PCR using primers of *frmA* N_{20}_F and pTargetF_R. The amplicon was digested with SpeI and self-ligated, resulting in pTargetF-*frmA* sgRNA. Then, the donor DNA cassette was excised from pUC19-*frmA*::*cat* by digestion with HindIII and EcoRI and inserted into the pTargetF-*frmA* sgRNA. The resulting plasmid was designated as pTargetF-*frmA*::*cat*.

The plasmid that can replace the *kdsD* gene in the genome of *Pseudomonas putida* NBRC 14164 with the *kan* gene was constructed as follows: The 1000-bp upstream region of *kdsD* and *kan* were amplified by PCR from the genome of *P. putida* NBRC 14164 and pK18mobsacB (National Institute of Genetics, Shizuoka, Japan) using the primer sets *kdsD* up_F/*kdsD* up_R and *kan*_F/*kan*_R, respectively. Amplicons were digested with SphI and ligated. The ligated fragment was amplified by PCR using primers *kdsD* up_F of *kan*_R. The amplicon was digested with NotI and BamHI and then ligated with a fragment consisting of *sacB* and *oriT* sequences, which was amplified from pK18mobsacB using the primers pK18mobsacB_F and pK18mobsacB_R, resulting in pMobsacB-*kdsD* up-*kan*. Subsequently, the 1000-bp downstream region of *kdsD* was amplified by PCR from the genome of *P. putida* NBRC 14164 using the primers *kdsD* down_F and *kdsD* down_R. The fragment was digested with SpeI and BamHI, followed by insertion into pMobsacB-*kdsD* up-*kan*. The resulting plasmid was designated as pMobsacB-*kdsD*::*kan*.

1.2 Gene replacement

The replacement of *frmA* with *cat* in the genome of *E. coli* MG 1655 was performed using pCas and pTargetF-*frmA*::*cat* as described previously (Okano et al., 2021). pCas is also a gift from Sheng Yang (Addgene plasmid #62225; http://n2t.net/addgene:62225; RRID:Addgene_62225) (Jiang et al., 2015). The resulting mutant was designated as *E. coli* Cm^R.

The replacement of *kdsD* with *kan* in *P. putida* NBRC 14164 was performed by the conjugational transfer of pMobsacB-*kdsD*::*kan*. The plasmid was first introduced into *E. coli* S17-1. The transformant was cultivated in LB medium containing 50 µg/mL of kanamycin overnight at 37°C. *P. putida* was also cultivated in LB medium overnight at 30°C. Next, 100 µL of *E. coli* culture and 500 µL of *P. putida* culture were mixed and centrifuged at 8,000 × *g* for 3 min. Then, the resulting pellet was suspended in 1 mL of LB medium, and 100 µL of portion was spread onto LB agar medium to induce the conjugational transfer of the plasmid from donor cells (*E. coli*) to recipient cells (*P. putida*). After colony formation, all colonies were suspended in 3 mL of LB medium and centrifuged at 8,000 × *g* for 3 min. Then, the cells were resuspended in 1 mL of LB medium, and 100 µL of which was spread onto LB agar medium supplemented with 50 µg/mL of kanamycin and 25 µg/mL of nalidixic acid to induce the integration of pMobsacB-*kdsD*::*kan* to the *kdsD* locus in the genome of *P. putida* and remove *E. coli* cells. The integration of pMobsacB-*kdsD*::*kan* into the upstream region of *kdsD* was confirmed by colony PCR using the primers *kdsD* up_F2 and *kan*_R (Supplementary Table S4). The integrant was cultivated in LB medium supplemented with kanamycin overnight at 37°C, and 100 µL of culture dilutant (100 times) was spread onto LB medium containing 10% (w/v) sucrose to induce plasmid excision from the genome and curing of the plasmid. The kanamycin-resistant mutant was selected and designated *P. putida* Neo^R because the mutant also showed resistance to neomycin.

2 Supplementary Figures and Tables

2.1 Supplementary Figures

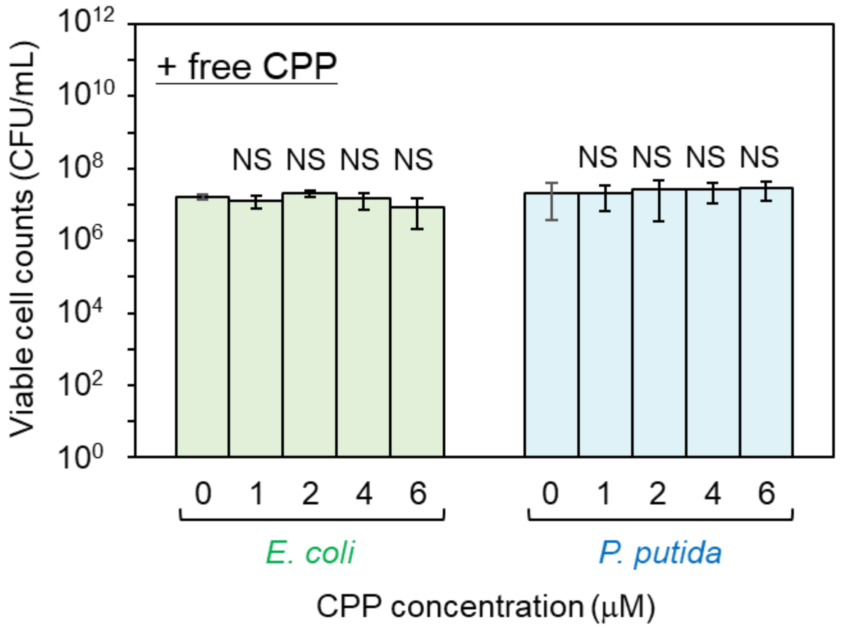

Supplementary Figure 1. Evaluation of the antibacterial activity of free CPP against *E. coli* and *P. putida*. CPP was added at various concentrations to the culture of *E. coli* and *P. putida*. After cultivation at 30°C for 24 h, the viable cell numbers were counted. Data bars represent mean ± standard deviation values of three independent experiments. The growth of both strains with the addition of free CPP was compared with that in nontreated condition. NS indicates that there was no significant difference in the *t*-test.

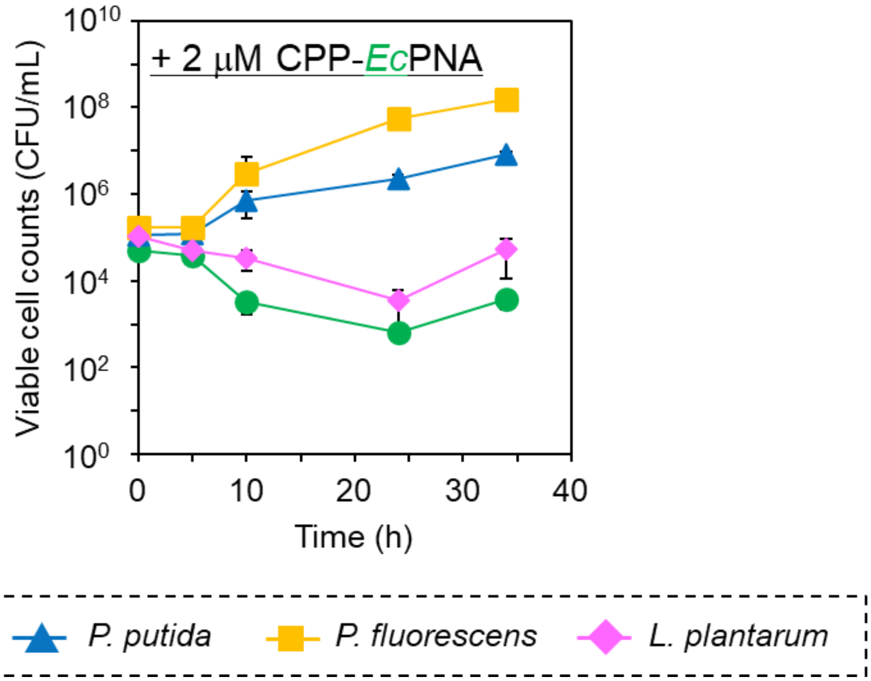

Supplementary Figure 2. Subtractive modification of the artificial bacterial consortium consisting of four bacterial species. *E. coli* (green circles), *P. putida* (blue triangles), *P. fluorescens* (orange squares), and *L. plantarum* (pink diamonds) were co-inoculated to M9 medium at 1.0×10^5 CFU/mL of each. The growth of four bacteria with 2 μM CPP-*Ec*PNA was shown in the figure. Data points represent mean ± standard deviation of three independent experiments.

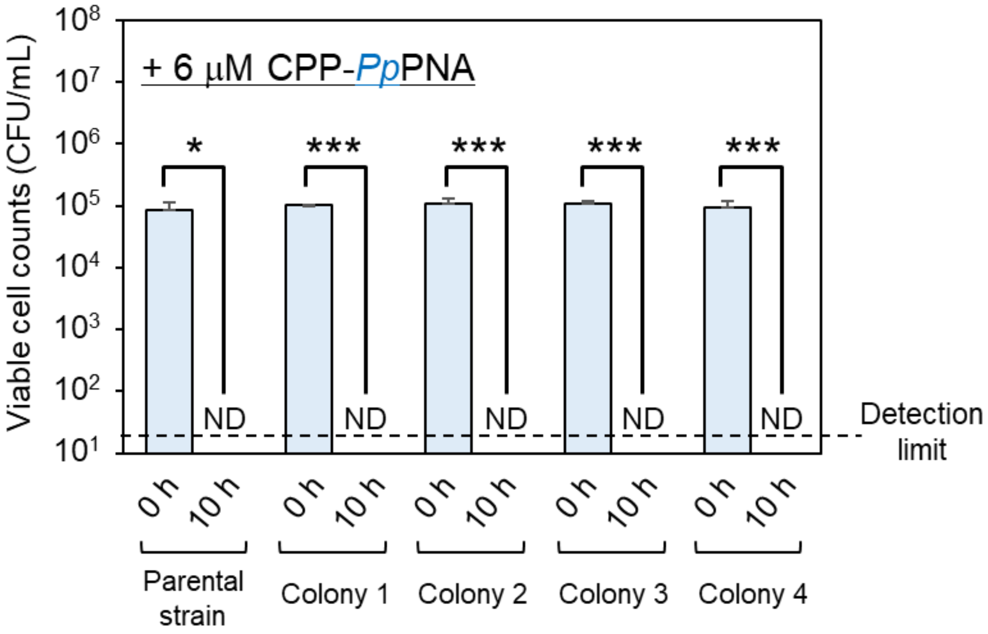

Supplementary Figure 3. Evaluation of the resistance of *P. putida* against CPP-*Pp*PNA. After 34 h of subtractive modification with adding 6 μM CPP-*Pp*PNA shown in Figure 3C, four colonies of *P. putida* were randomly picked up. They were inoculated to M9 medium at 1.0×10^5 CFU/mL and their resistance to 6 μM CPP-*Pp*PNA was compared with that of the parental strain. Dashed line indicates the detection limit of viable cell counts (20 CFU/mL) and ND indicates that no colony was detected. Data bars represent mean ± standard deviation values of three independent experiments. Asterisks and triple-asterisks indicate p values are less than 0.05 and 0.005 in the t-test, respectively.

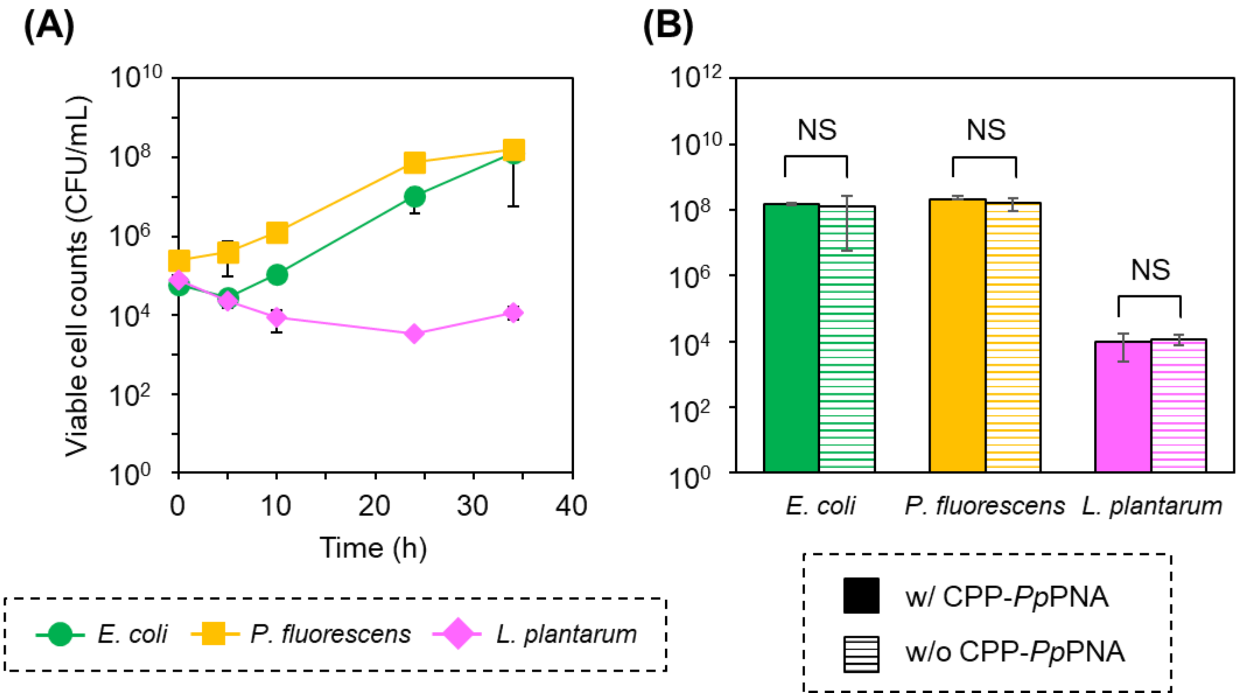

Supplementary Figure 4. Cultivation of the artificial bacterial consortium without *P. putida*. (A) *E. coli* (green circles), *P. fluorescens* (orange squares), and *L. plantarum* (pink diamonds) were co-inoculated to M9 medium at 1.0 × 10^5 CFU/mL of each. (B) Comparison of the final CFU (horizontal bars) with the four-species mixed culture with 6 μM CPP-*Pp*PNA addition (solid bars; result from Figure 3C). Data points and bars represent the mean ± standard deviation values in three independent experiments. NS indicates that there was no significant difference in the *t*-test.

2.2 Supplementary Tables

Supplementary Table S1. Growth tests under various culture conditions

Strain	LB + 30 μg/L chloramphenicol	LB + 50 μg/L neomycin	LB + 50 μg/L streptomycin	MRS (pH = 6.0)	37°C
E. coli Cm^R	+	−	−	−	+
P. putida Neo^R	−	+	−	−	−
P. fluorescens	+	−	+	−	−
L. plaantarum	−	−	−	+	+

Supplementary Table S2. Sequences of PNA binding site in various *Pseudomonas* species

Strain	Target sequence in *ftsZ* [a,b]	Target sequence in *acpP* [a,b]	Accession number of sequence
P. aeruginosa PAO1	(+4) TTTGAACTGG (+13)	(-6) CAAGGTATGAG (+6)	GenBank: CP129519.1
P. mendocina CPS5	(+4) TTCGAACTAG (+13)	(-6) CAAGGTATGAG (+6)	NCBI RefSeq: NZ_CP060288.1
P. stutzeri F2a	(+4) TTCGAACTCG (+13)	(-6) CAAGGTATGAG (+6)	GenBank: AP024722.1
P. brassicacearum LBUM300	(+4) TTCGAACTCG (+13)	(-6) CAAGGTATGAG (+6)	NCBI RefSeq: NZ_CP012680.1
P. fluorescens NBRC 14160	(+4) TTCGAACTCG (+13)	(-6) CAAGGTATGAG (+6)	NCBI RefSeq: NZ_BDAA00000000.1
P. putida NBRC 14164	(+4) TTCGAGCTCG (+13)	(-6) CTAGGTATGAG (+6)	GenBank: AP013070.1

[a] Numbers in parentheses indicate the location from translation start site

[b] Sequence having mismatch with the *P. aeruginosa* sequence were shown in red

Supplementary Table S3. Plasmids used in this study

Plasmid	Relevant description	Source or reference
Plasmids		
Escherichia coli		
pUC19	pMB1 *ori*, *bla*	Takara Bio
pUC19-Δ*frmA*	pMB1 *ori*, *bla*, donor DNA for deleting *frmA*	This study
pHLA	source of *cat* gene	Narita et al., 2006
pUC19-*frmA*::*cat*	pMB1 *ori*, *bla*, donor DNA for replacing *frmA* with *cat*	This study
pTargetF	pMB1 *ori*, *aadA*	Jiang et al., 2015
pTargetF-*frmA* sgRNA	pMB1 *ori*, *aadA*, P_{J23119}-*frmA*-sgRNA	This study
pTargetF-*frmA*::*cat*	pMB1 *ori*, *aadA*, P_{J23119}-*frmA*-sgRNA, donor DNA for replacing *frmA* with *cat*	This study
pCas	*rep101*(Ts), *kan*, P_{cas}-*cas9*, P_{araB}-*Red*, *lacI*^{q}, P_{trc}-sgRNA-*pMB1*	Jiang et al., 2015
Pseudomonas putida		
pK18mobsacB	*oriT* (RP4), *sacB*, *lacZ*α, *kan*	National Institute of Genetics
pMobsacB-*kdsD*::*kan*	*oriT* (RP4), *sacB*, *lacZ*α, *kdsD*::*kan* cassette	This study

Supplementary Table S4. Sequence of oligonucleotide primers used in this study

Primer	Sequence (5'-3') [a]
frmA up_F	CCCAAGCTTTGATTCCTTCTGCCGCC
frmA up_R	ACGCGTCGACGAGCTCCTCTCGCTCTTCCTCAATATGGTAATAG
frmA down_F	ACGCGTCGACTTTCCCGCAGGTTTACCCC
frmA down_R	CAGGAATTCGTTGCCGGAAAATGATGCATC
*cat*_F	AACGAGCTCGTTACAGTAATATTGACTTTTAAAAAAGGATTG
*cat*_R	ACGCGTCGACTTATAAAAGCCAGTCATTAGGCCTATC
frmA N_{20}_F	GGACTAGTGCGCTGGAAAGTGCGCACCGGTTTTAGAGCTAGAAATAGCAA GTTAAAATAAGG
pTargetF_R	GGACTAGTATTATACCTAGGACTGAGCTAGC
kdsD up_F	ATAGCGGCCGCGGGCCGTCCGGGTCG
kdsD up_F2	CAGGAAAGTGGAAGGGAACC
kdsD up_R	ACATGCATGCGTCGAGAAAGCAATGCCTGATG
*kan*_F	ACATGCATGCAGGAAGCGGAACACGTAG
*kan*_R	CGCGGATCCACTAGTTCAGAAGAACTCGTCAAGAAGG
pK18mobsacB_F	ATCGCGGCCGCAGCTGTTTCCTGTGTGAAATTG
pK18mobsacB_R	CGCGGATCCGCGGGACTCTGGGGTTC
kdsD down_F	GGACTAGTGGAGCGATGGAATGAACCAG
kdsD down_R	CGCGGATCCTTGCTCGGTCTGCGC

[a] Restriction enzyme sites are underlined

[b] N_{20} sequence is double underlined

3 References

Jiang, Y., Chen, B., Duan, C., Sun, B., Yang, J., and Yang, S. (2015). Multigene editing in the *Escherichia coli* genome via the CRISPR-Cas9 system. *Appl. Environ. Microbiol*. 81 (7), 2506–2514. doi:10.1128/AEM.04023-14

Narita, J., Okano, K., Tateno, T., Tanino, T., Sewaki, T., Sung, M.-H. et al. (2006). Display of active enzymes on the cell surface of *Escherichia coli* using PgsA anchor protein and their application to bioconversion. *Appl. Microbiol. Biotechnol.* 70 (5), 564–572. doi:10.1007/s00253-005-0111-x

Okano, K., Sato, Y., Hizume, T., and Honda, K. (2021). Genome editing by miniature CRISPR/Cas12f1 enzyme in *Escherichia coli*. *J. Biosci. Bioeng.* 132 (2), 120–124. doi:10.1016/j.jbiosc.2021.04.009
